# Metabolic-Associated Fatty Liver Disease (MAFLD), Diabetes, and Cardiovascular Disease: Associations with Fructose Metabolism and Gut Microbiota

**DOI:** 10.3390/nu14010103

**Published:** 2021-12-27

**Authors:** Karolina Drożdż, Katarzyna Nabrdalik, Weronika Hajzler, Hanna Kwiendacz, Janusz Gumprecht, Gregory Y. H. Lip

**Affiliations:** 1 Department of Internal Medicine, Diabetology and Nephrology in Zabrze, Faculty of Medical Sciences in Zabrze, Medical University of Silesia, 40-055 Katowice, Poland; karolina.drozdz92@gmail.com (K.D.); hkwiendacz@sum.edu.pl (H.K.); jgumprecht@sum.edu.pl (J.G.); gregory.lip@liverpool.ac.uk (G.Y.H.L.); 2 Liverpool Centre for Cardiovascular Science, University of Liverpool and Liverpool Heart & Chest Hospital, Liverpool L14 3PE, UK; 3 Doctoral School, Department of Pediatric Hematology and Oncology in Zabrze, Faculty of Medical Sciences in Zabrze, Medical University of Silesia, 40-055 Katowice, Poland; hajzlerweronika@gmail.com; 4 Department of Clinical Medicine, Aalborg University, 9100 Aalborg, Denmark

**Keywords:** metabolic–associated fatty liver disease, diabetes mellitus, cardiovascular disease, fructose, microbiota

## Abstract

Non-alcoholic fatty liver disease (NAFLD) is an increasingly common condition associated with type 2 diabetes (T2DM) and cardiovascular disease (CVD). Since systemic metabolic dysfunction underlies NAFLD, the current nomenclature has been revised, and the term metabolic-associated fatty liver disease (MAFLD) has been proposed. The new definition emphasizes the bidirectional relationships and increases awareness in looking for fatty liver disease among patients with T2DM and CVD or its risk factors, as well as looking for these diseases among patients with NAFLD. The most recommended treatment method of NAFLD is lifestyle changes, including dietary fructose limitation, although other treatment methods of NAFLD have recently emerged and are being studied. Given the focus on the liver–gut axis targeting, bacteria may also be a future aim of NAFLD treatment given the microbiome signatures discriminating healthy individuals from those with NAFLD. In this review article, we will provide an overview of the associations of fructose consumption, gut microbiota, diabetes, and CVD in patients with NAFLD.

## 1. Introduction

**Figure 1:**
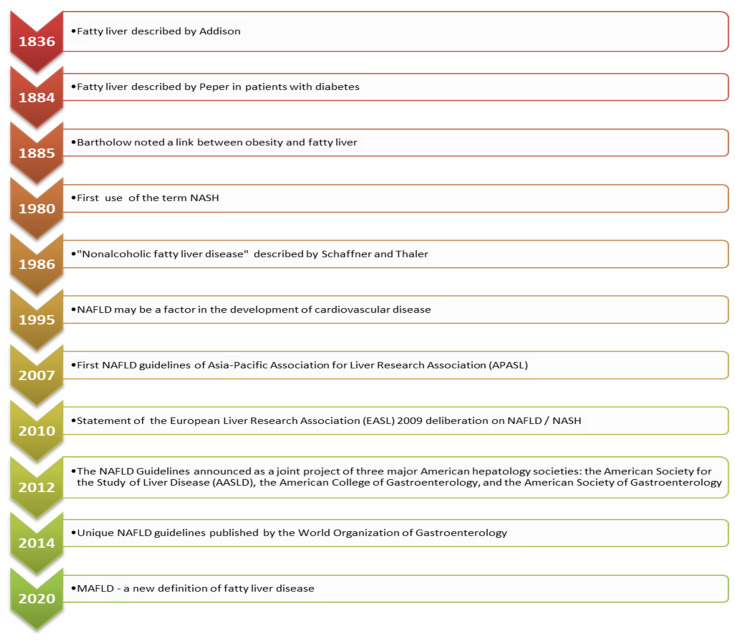
Fatty liver disease timeline [1,2,3,4,5,6,7,8].

In 1986, the term nonalcoholic fatty liver disease (NAFLD) was proposed by Schaner and Thaler [1]. Almost 10 years later, Leonardo et al. hypothesized that NAFLD could be associated with cardiovascular disease (CVD) [2], and soon after, the first NAFLD guidelines emerged [3,4,5]. The most relevant events in the NAFLD history [1,2,3,4,5,6,7,8] are presented in Figure 1.

The definition of NAFLD combines the presence of steatosis in more than 5% of hepatocytes and metabolic risk factors, especially obesity and T2DM, and exclusion of excessive alcohol consumption defined as ≥30 g per day for men and ≥20 g per day for women, or other chronic liver diseases [9]. It should be noted that the liver is a primary organ for lipid and glucose homeostasis and is the focus of cardiometabolic disease. In 2020, fatty liver was redefined from negative (absence of excessive alcohol consumption and other known causes of liver disease) to a more positively stated metabolic associated fatty liver disease (MAFLD) [10].

**Figure 2:**
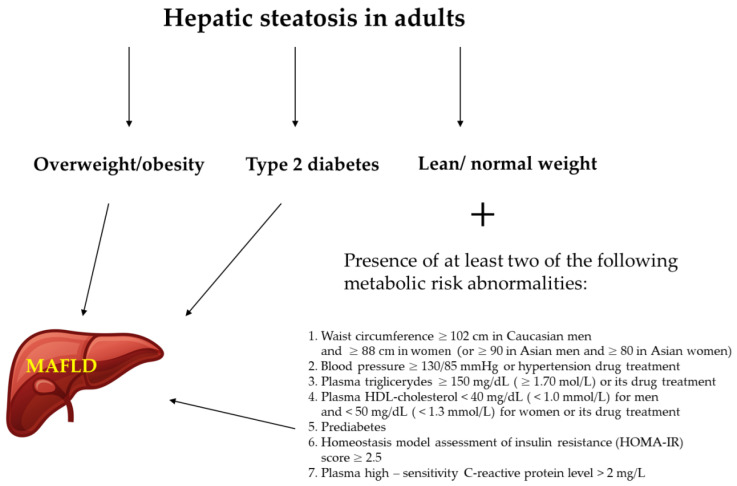
Diagnostic criteria for MAFLD (metabolic dysfunction–associated fatty liver disease) [12].

The latter definition of MAFLD is based on the presence of hepatic steatosis and at least one other condition such as overweight/obesity, T2DM, or metabolic abnormalities with no additional exclusion criteria [10,11]. Metabolic abnormalities included in the definition cover at least two features from the following: increased waist circumference, arterial hypertension, hypertriglyceridemia, low high-density cholesterol (HDL-C), prediabetes, insulin resistance, and subclinical inflammation [10] (Figure 2). This new definition underlines the importance of cardiometabolic risk factors contributing to liver disease even among patients with other liver diseases and who drink alcohol [10]. Although it should be noticed that although the new definition has been proposed by a panel of international experts from 22 countries, this new nomenclature is not yet accepted by the American Association for the Study of Liver Disease and the European Association for the Study of Liver Disease.

Diabetes mellitus is a heterogeneous group of disorders that results in an increase in blood glucose concentration, yet the pathophysiological processes underlying type 1 and type 2 of the disease differ. Insulin resistance, which is tightly linked to T2DM, is not always present in type 1 of the disease, thereby explaining the different prevalence of NAFLD in those two subpopulations of patients [11].

NAFLD has a high prevalence in the general population, varying from 13.5% in Africa to 31.8% in the Middle East [13]. It is diagnosed in 47.3–63.7% of patients with T2DM and up to 80% of people with obesity [14,15]. Liver failure related to NAFLD is the second leading cause of liver transplantation in the western world [15]. The high prevalence of NAFLD is driven mainly by an unhealthy lifestyle, including dietary factors such as high levels of saturated fats, cholesterol, or fructose [16].

Consumption of fructose has increased over the last century mainly due to the use of high-fructose corn syrup [17]. This phenomenon has been studied in the context of liver disease, obesity, and diabetes, where it promotes hepatic de novo lipogenesis, leading to lipid accumulation in the liver and insulin resistance [18,19,20]. A new perspective on NAFLD emerged during the last decade when scientists additionally focused on the relationship of microbiota with metabolic disease and its relation to NAFLD, where microbiome signatures discriminate healthy individuals from those with NAFLD [21].

While there is the perception that NAFLD is a benign liver condition, it is the second most common cause of end-stage liver disease [22] and the second cause of primary liver cancer in patients waiting for liver transplants in the US [13] and Europe [23]. Even more importantly, the leading cause of death among patients with NAFLD is not associated with the liver itself but with CVD [24].

Since MAFLD is a new term and has been seldom used in published studies, for this review article, we have summarized the current state of knowledge regarding NAFLD and its association with fructose consumption, microbiota, diabetes, and CVD.

## 2. NAFLD and Fructose

Fructose’s use in foods was limited until the late 1960s due to its high price [25]. Since then, it has become freely available and has been shown to exert a positive effect in the treatment of diabetes since fructose does not require insulin to be metabolized and has no effect on fasting blood glucose levels and urinary glucose excretion [26,27]. Nowadays, the way scientists look at fructose has changed, and it is now known as a risk factor in the development of obesity and several metabolic disturbances, NAFLD, among others [19].

Fructose is a major dietary monosaccharide that occurs naturally in ripe fruits, honey, and in small amounts in some vegetables such as carrot, onion, paprika, and sweet potato. It also comes in industrially manufactured foods because it is a main ingredient in the most widely used sweeteners like disaccharide sucrose (table sugar, composed of one glucose molecule and one fructose) and high fructose glucose syrup (mixture of fructose with sucrose or glucose) [18,27].

Fructose, in contrast to glucose, is almost totally cleared from circulation by the liver with the use of glucose transporter type—5 (GLUT 5). A large amount of acetyl-CoA is produced following fructose uptake because fructose clearance omits glycolysis, which is the rate-limiting step in acetyl-CoA production [28]. Some acetyl-CoA is used for ATP production, but the excess amount is used for de novo lipogenesis, which is one of the mechanisms proposed for how consuming fructose leads to NAFLD [28,29]. Based on the results of studies on animals and humans with fructose overfeeding, high fructose consumption (25% of energy requirement) may increase visceral adiposity, postprandial hypertriglyceridemia, and insulin resistance by acting on de novo lipogenesis [30].

However, it is not only lipogenesis; other hepatotoxic effects are exerted by fructose, namely inducing an increase in oxidative stress [31]. Fructose is able to directly generate reactive oxygen species (ROS) and lead to hepatocellular damage through protein fructosylation [32]. Because of the increased consumption of processed foods, fructose consumption has increased dramatically, by 30% over the last 40 years and by 500% over the last century [33]. Also, there was more than a 40% increase in the intake of sugar-sweetened beverages (SSB) from 1990 to 2016 [34]. Because free sugars consumption has a proven association with metabolic diseases and cancer, it has since been recommended by the World Health Organization (WHO) to decrease its consumption to less than 10% of total daily energy intake [35].

Numerous animal and human studies have revealed the association of a close relationship between the consumption of fructose and the development of NAFLD (Table 1).

In a systematic review and meta-analysis of observational studies involving 4639 individuals, SSB consumption led to a 53% increased risk of developing NAFLD in comparison to participants who did not ingest SSB [46]. Another analysis of 12 studies with 35,705 individuals showed that higher consumption of SBB was associated with a 40% increase in the incidence of NAFLD [47]. Other studies also reported increased risk of NAFLD associated with consumption of SBB [36,37,38,39,40,43], yet some of the meta-analyses indicate the increase of intrahepatocellular lipids only under conditions of hypercaloric diet [41,42]. An interesting observation is that the association between SBB intake and liver fat may be independent of BMI [45]. One small RCT concluded that there was no significant change in hepatic fat or body weight in the groups consuming fructose or glucose beverages only; however, reduction of fructose led to improvement of several factors related to CVD [44]. What is also interesting to note is that fructose consumption influences the composition and function of gut microbiota in a way that promotes the development and progression of NAFLD [48].

## 3. NAFLD and Gut Microbiota

The pioneering studies using germ-free mice and gut microbiota transfer related to the association of gut microbiome with metabolic diseases revealed a contribution of gut microbiota to weight gain and metabolic alterations [49]. During the last decade, there has been a growing body of evidence demonstrating the contribution of the gut microbiome to the pathogenesis of NAFLD [50,51,52]. In general, dysfunction of the gut–liver axis caused by bacterial proliferation in the intestine, alteration of the intestinal permeability, and intestinal dysbiosis have a large influence on the development and progression of NAFLD [11]. Initial studies demonstrated that genetically modified mice (modification in the inflammasome pathway) were prone to develop NASH when co-housed with wild-type mice, leading to the development of liver steatosis and inflammation in wild-type mice as a consequence of microbiota sharing through coprophagia [53].

Fecal microbiota transfer from patients with NASH to germ-free mice causes hepatic steatosis and inflammation in these animals [54]. On the other hand, the outcomes of animal studies cannot be directly extrapolated to humans (e.g., mice do not develop the whole range of steatosis stages seen in humans, and mice microbiota itself differs from humans) [55,56].

Human studies have been based on comparisons of gut microbiota between patients with NAFLD, NASH, and cirrhosis and individuals with a healthy liver performed to demonstrate gut microbiota signatures in these pathological conditions [57]. Gut microbiota, and specifically, the gut–liver axis’ role in the pathogenesis of NAFLD, has been explored in up-to-date studies, but this relationship is still poorly defined. Nevertheless, gut microbiome signatures in NAFLD, NAFLD fibrosis, and cirrhosis could become non-invasive diagnostic biomarkers for liver disease diagnosis [21].

The gut–liver axis is an association between gut microbiota and the liver. The interaction is conducted through the portal vein, which transports products from the gut to the liver and, in return, bile and antibodies from the liver to the intestine [58]. An important determining health factor seems to be a mucosal barrier comprised of intestinal epithelial cells. Its permeability and mucus composition are derived from gut microbiota and the presence of immune cells [58,59]. Increased permeability of the intestinal mucosal barrier and unfavorable changes in gut microbiota compositions are possible factors in the development and progression of NAFLD [58,59]. Dysfunction of gut microbiota results in the production of PAMPs (pathogen-associated molecular patterns), and increased permeability of the mucosal barrier leads to increased inflammation in the liver and the development and progression of liver disease [58,59,60]. Studies have shown a lower diversity of microbiota in patients with NAFLD compared to healthy controls [61,62].

Abnormalities of gut microbiota composition in stools of patients with NAFLD have been highlighted in a meta-analysis showing increased abundance of Escherichia, Prevotella, and Streptococcus and decreased abundance of Coprococcus, Feacalibacterium, and Ruminococcus [63]. When patients with liver fibrosis and patients with severe steatosis without fibrosis were compared, fecal Clostridium was significantly decreased in patients with liver fibrosis and was negatively associated with liver stiffness measurement (LSM) and myosteatosis [64]. Patients with liver fibrosis had increased fecal abundance of Escherichia and Shigella compared to patients with severe steatosis without fibrosis [64]. In another study in patients with NASH, there were increased levels of Escherichia and Shigella comparing patients with liver biopsy results as F0 (absence of fibrosis) or F1 (perisinusoidal or periportal fibrosis) [62].

NAFLD also results in increased fecal ester volatile organic compounds (VOC) and the presence of higher concentrations of fecal propionate and isobutyric acid and serum 2-hydroxybutyrate and L-lactic acid [65,66]. VOC is the result of gut microbiota substrate fermentation and is considered a potential marker of intestinal dysbiosis [67]. The results of the mentioned studies are summarized in Table 2.

## 4. Treatment of NAFLD with Microbiome Alterations

The aforementioned associations between NAFLD and gut microbiota have resulted in studies investigating the effects of microbiome alternation with probiotics, prebiotics, synbiotics, or fecal microbiota transplantation (FMT) on the clinical course of NAFLD with promising results [68,69,70,71,72].

According to the definitions formulated by the WHO and Food and Agriculture Organization of the United Nations (FAO), probiotics are live strains of strictly selected microorganisms which, when administered in adequate amounts, confer a health benefit on the host [73]. Prebiotics are described as a nonviable food component that confers a health benefit on the host associated with modulation of microbiota [74]. Synbiotics are the combination of synergistically acting probiotics and prebiotics; their role is the improvement of the survival of probiotic microorganisms in the gastrointestinal tract [75].

In this review article, we focus on the meta-analysis of RCT (randomized controlled trials), showing that probiotic/synbiotic therapy results in improving liver enzymes’ activity and/or reduced steatosis/fibrosis in patients with NAFLD [68,69,70,71]. Moreover, treatment with probiotics decreases levels of CRP (C-reactive protein) and TNF-α (tumor necrosis factor α), suggesting the reduction of inflammation and playing an important role in NAFLD pathogenesis [68,70,72,76]. The studies related to synbiotics in patients with NAFLD are limited. In a recent RCT, there was a reduction in steatosis and improved liver enzyme changes observed in patients treated with *bifidobacterium animalis* and insulin [77]. In another study, the use of synbiotics positively influenced inflammatory markers in NAFLD [78].

The role of FMT in patients with NAFLD has limited RCT evidence. FMT was first proven to be a good treatment method of antibiotic-resistant *clostridium difficile* infection in 2013 [79] and soon after was tested in other diseases, including metabolic diseases [80]. In an RCT including 21 patients undergoing allogenic or autologous FMT, the procedure was not associated with an improvement in insulin resistance nor hepatic proton density fat fraction but had the potential to lower small intestinal permeability [81]. In the study by Witjes et al., allogenic FMT using lean vegan donors modified gut microbiota composition with favorable changes in plasma metabolites and steatohepatitis [82]. The aforementioned studies are summarized in Table 3.

## 5. NAFLD and T2DM

According to epidemiological data, the overall prevalence of NAFLD among patients with T2DM is 55.5%, and NASH is 37.3%; also, 17% of T2DM patients who underwent liver biopsy have advanced fibrosis [14]. Current guidelines emphasize the role of screening for diabetes in patients diagnosed with NAFLD and vice versa [9,83]. NAFLD itself increases the risk of T2DM incidence [84,85,86], and the risk of the new-onset T2DM is doubled in patients with NAFLD [87]. Moreover, 25% of patients with NAFLD also have T2DM [88].

NAFLD and insulin resistance are interconnected, and therefore the development of prediabetes and diabetes is the most direct consequence of them at the extrahepatic level [11,89]. Particularly, the coexistence of NAFLD and T2DM worsens the course of both conditions since this relationship is bidirectional [90,91,92]. One meta-analysis found that the risk of T2DM is greater in patients with advanced NAFLD with fibrosis [84], and in another meta-analysis evaluating whether NAFLD predicted T2DM, NAFLD predicted the risk of T2DM independent of age and obesity, irrespective of the NAFLD diagnosis method (ultrasonography or elevated liver enzymes) [85]. Moreover, this observation was also applicable to patients with prediabetes and NAFLD, where the incidence of T2DM was higher than in patients with prediabetes without NAFLD [86]. When different types of diabetes were analyzed, the prevalence of NAFLD was low in patients with type 1 diabetes mellitus but high in T2DM patients in whom NAFLD was associated with increasing BMI, triglycerides, ALT (alanine aminotransferase) serum concentration, and decreasing adiponectin concentration [93]. Surprisingly, NAFLD occurred more often in T2DM patients not treated with insulin than in patients treated with insulin [93].

In another cohort study of 10,141 participants, future diabetes mellitus risk could be modified with time by changes in NAFLD status where resolution of NAFLD could diminish the risk of diabetes onset. On the other hand, the development of NAFLD raised the risk of developing diabetes [94]. Current evidence suggests that the magnitude of risk of incident T2DM mirrors the severity of NAFLD, especially with the severity of liver fibrosis [95].

It is important to distinguish between simple steatosis, which does not progress to advanced fibrosis, and non-alcoholic steatohepatitis (NASH), which can lead to hepatocellular carcinoma. Indeed, NASH is characterized by fat accumulation, inflammation and necrosis, ballooning of the cells, and different stages of liver fibrosis, up to cirrhosis. The only method to differentiate between steatosis and NASH is liver biopsy. Masarone et al. performed this invasive procedure in 215 patients with elevated transaminases and metabolic syndrome or T2DM. The prevalence of NAFLD in patients with metabolic syndrome was 94.82% and was present in all the patients with T2DM. NASH was found in 58.52% of participants with metabolic syndrome and 96.82% of T2DM patients. According to the authors, one can assume that patients with T2DM have NASH. As insulin resistance is of crucial importance in the pathophysiology of both T2DM and NASH, NASH may be one of the early complications of T2DM [96].

Some studies have reported that a reduction in the T2DM incidence [97] and the clinical manifestation of atherosclerosis [98] could be achieved by the pharmacological eradication of the hepatitis C virus with direct-acting antiviral drugs [99], caused by the reduction of insulin resistance and improving various HCV-induced glucose homeostasis mechanisms [97,98].

The strong link between T2DM and NAFLD was underlined in 2020 by an international panel of experts who proposed the new term MAFLD instead of NAFLD [10]. The results of the studies described above are presented in Table 4.

## 6. Treatment of NAFLD with Antidiabetic Drugs

Currently, there is no single, independent of the presence or absence of T2DM, pharmaceutical treatment for NAFLD that has been approved by international guidelines [9]. The frequent coexistence of diabetes and NAFLD and complex pathogenesis of these two metabolic diseases result in the growing interest in antidiabetic drugs used in the treatment of NAFLD.

The best documented direct beneficial effect remains assigned to pioglitazone [100,101,102,103], yet incretin drugs [104,105,106,107,108,109,110,111,112,113,114,115,116,117,118,119,120,121,122] seem to present favorable action via indirect modifications of metabolic risk factors and direct course of action with the most promising combination of glucagon-like peptide-1 receptor agonist (GLP-1 RA) and agonist of glucose-dependent insulinotropic polypeptide (GIP) [123].

Current guidelines state that pharmacotherapy is reserved for patients with NASH and for patients at high risk of disease progression. Among the antidiabetic drugs recommended, only pioglitazone is used for the treatment of NASH with insulin resistance (evidence level A, strength 2) [9,124]. Older studies showed that NASH treatment with pioglitazone caused histological improvement of hepatic steatosis and fibrosis [100,101,102,103]. In an RCT analyzing patients with T2DM or prediabetes and NASH, pioglitazone usage for 18-months resulted in a reduction of fibrosis score and hepatic triglyceride content and improved insulin sensitivity of adipose tissue, liver, and muscles [103]. In a recent meta-analysis, pioglitazone treatment in patients with NAFLD with or without T2DM caused significant reductions of ALT and AST (aspartate aminotransferase) levels [104].

Two relatively new antidiabetic drug classes, namely SGLT-2i (sodium-glucose cotransporter type-2 inhibitors) [105,114,115,116,117] and GLP-1 RA [104,106,107,108,109,110,111,112,118,119,120,121,122] show promise in the treatment of NAFLD/NASH patients with T2DM and have been added to the revised guidelines from the year 2020 (evidence level C, strength 2) [124]. Revised guidelines indicate that both SGLT-2i and GLP-1 RA improved liver enzymes and histological findings [124]. The latest meta-analyses of the effects of SGLT-2i [113] and GLP-1 RA [125] in NAFLD patients with T2DM, respectively, were published. In the study related to SGLT-2i, canagliflozin improved liver function parameters while dapagliflozin was better in improving glycemia and insulin sensitivity [113]. In the meta-analysis of GLP-1-RA, there was strong evidence that GLP-1 RA improved liver function and histology [125].

An upcoming drug of huge interest is a dual GIP and GLP-1 RA agent (tirzepatide), which is under investigation. It is being administered once a week in the therapy of T2DM patients with NASH and fibrosis [123]. Treatment with this drug for 26 weeks compared to dulaglutide and placebo resulted in a significant reduction in NASH-related biomarkers, an increase in adiponectin, and a greater reduction of ALT, compared to dulaglutide treatment [123]. A published network meta-analysis of RCTs [126] assessed the effectiveness of antidiabetic medications for T2DM as potential therapeutic agents for NAFLD, comparing SGLT-2i, GLP-1 RA, PPARγ (peroxisome proliferator-activated receptor) agonists, biguanides, sulfonylureas, and insulin. This showed that GLP-1 RA and SGLT-2i led to reductions in BMI, fibrosis, and steatosis, with SGLT-2i being the best treatment for reducing low-density lipoprotein cholesterol (LDL-C) and rising HDL-C. Studies related to the SGLT-2i, GLP-1 RA, and dual GIP and GLP-1-RA use in the treatment of NAFLD are summarized in Table 5.

## 7. NAFLD and Cardiovascular Disease

Both NAFLD and CVD are highly prevalent and associated with metabolic disturbances; hence they frequently coexist [127]. This causal relationship may be due to shared common pathophysiological pathways, which include low-grade inflammation, oxidative stress, and insulin resistance [128].

NAFLD is linked with different manifestations of CVD, including subclinical atherosclerosis, overt atherosclerosis, and cardiovascular events and deaths [24,129,130,131]. Hence, both the American Association for the Study of Liver Disease and the European Association for the Study of the Liver suggest cardiovascular screening in patients with NAFLD [9,132]. In relation to the association of NAFLD with the risk of major adverse cardiovascular events (MACE), several meta-analyses and cohort studies show that in patients with NAFLD, the risk of MACE is increased, independent of other cardiovascular risk factors or the extent of coronary disease [133,134].

One meta-analysis from the year 2021 deserves special attention because it was performed among people with histologically confirmed NAFLD who did not present with CVD at baseline (10,422 participants) and were prospectively followed–up for a median of 13.6 years [134]. This study showed that patients with biopsy-proven NAFLD who were matched to controls had a higher incidence of MACE, which included ischemic heart disease, congestive heart failure, and cardiovascular mortality [134]. Moreover, the rates of incident MACE increased progressively with worsening NAFLD severity.

There are also studies that indicated that CVD risks in patients with NAFLD who are non-obese [135] or non-overweight [136] were also increased. Additionally, in obese patients with NAFLD, liver fat content (LFC) >10% was also a predictor of subclinical atherosclerosis [135].

One retrospective, “real world” cohort study aimed to describe the CVD burden and mortality in patients with NAFLD during 14-years follow-up observation following hospital discharge, showing that in patients with non–cirrhotic NAFLD, the condition was associated with increased overall mortality [137]. In contrast, other studies have stated that NAFLD was not correlated with CVD mortality [138,139,140] nor an increased risk of acute myocardial infarction or stroke [141].

Due to the recent change in nomenclature of fatty liver disease (NAFLD to MAFLD), the prevalence of fatty liver disease and associated CVD risk required re-evaluation, taking into account each of these definitions separately [142]. An analysis of 9,584,399 participants aged 40–64 from the National Health Database revealed that MAFLD and NAFLD, independent of the definition used, increased the risk of CVD events [142]. Furthermore, CVD seems to be increased when NAFLD coexists with T2DM. A meta-analysis of 11 studies, including cross-sectional and cohort studies, indicated that the risk for CVD in T1DM and T2DM patients with NAFLD was increased two-fold compared to patients without NAFLD [143]. In T2DM patients with NAFLD, the occurrence of coronary artery disease (CAD) was more prevalent than in T2DM patients without NAFLD [144,145,146]. Interestingly, patients diagnosed with NAFLD before T2DM diagnosis showed a higher prevalence of CAD and hypertension when compared to groups of patients with T2DM without NAFLD and T2DM diagnosed before NAFLD [144]. Additionally, T2DM patients with NAFLD had a higher prevalence of hypertension, obesity, higher hemoglobin A1c (HbA1c) levels, higher triglyceride, lower HDL-C concentration, and higher mean carotid IMT (intima-media thickness) [145].

On the other hand, another study concerning atherosclerotic lesions in T2DM patients found no significant difference in carotid IMT between T2DM patients with and without NAFLD [147]. The same study demonstrated that the prevalence of carotid and lower limb atherosclerotic plaque and stenosis was higher in T2DM patients with NAFLD compared to T2DM patients without NAFLD [147]. A study by Kim et al. divided T2DM patients into two groups: insulin-resistant and insulin-sensitive, whereby mean carotid IMT was higher in subjects with both insulin resistance and NAFLD than in insulin-sensitive patients with or without NAFLD [148]. T2DM patients with co-existing NAFLD also have an increased risk of PAD (peripheral artery disease) defined in this study as ABI (ankle-brachial index) < 0.90 on either side [149].

While an association between NAFLD and T2DM microvascular complications seems plausible, the number of studies on this topic is limited and inconclusive [144,150,151,152,153,154]. In 2008, Targher et al. performed one of the first studies evaluating the associations between NAFLD and both chronic kidney disease (CKD) and retinopathy in a large cohort of 2103 patients with T2DM and found that NAFLD was independently associated with an increased prevalence of CKD and retinopathy [151]. In the Valpolicella Heart Diabetes Study, among 1760 outpatients with T2DM who had normal kidney function at baseline, those with NAFLD had an independently increased risk of incident CKD over a follow-up period of 6.5 years [155]. These initial observations have been confirmed in further clinical studies and meta-analysis of observational studies [152,153], and moreover, it was shown that NAFLD increases the risk of diabetic neuropathy [156]. However, there are also studies that do not show such a relationship [144,150,154]. Results of the relevant studies are included in Table 6.

It is well established that patients with DM compared to people without carbohydrate disorders have a higher risk of heart failure and CVD. There is a term “diabetic cardiomyopathy”, deteriorating patient’s prognosis and being described as a form of heart disease occurring in diabetic patients, which causes significant structural as well as functional changes in the myocardium. There is a common pathophysiological mechanism of diabetic cardiomyopathy and NAFLD, namely insulin resistance [157]. It results in an increase in lipogenesis in the liver and adipose tissue’s lipolysis inhibition [89]. Insulin resistance also causes a decrease in the concentration of an insulin-sensitizing adipokine called adiponectin. Physiologically, adiponectin plays an important role in hepatoprotection by modifying free fatty acids metabolism, gluconeogenesis, and lipogenesis. Moreover, by reducing the number of proinflammatory cytokines and promoting the proliferation of hepatic stellate cells, it prevents liver fibrosis. In NAFLD, there are a couple of mechanisms (including dysfunction in adipokine production, increase in oxidative stress reactions, and general proinflammatory state) that influence the atherosclerotic plaque formation and its progression, therefore, leading to the increase of cardiovascular risk [158].

## 8. Conclusions

MAFLD is a new clinical definition for fatty liver disease, which shifts NAFLD from a disease of exclusion to one of inclusion, where the pathogenic processes originate from underlying metabolic dysfunction. Because MAFLD is not widely used terminology in the scientific literature, most published data focus on NAFLD. The latter as an epidemic is tightly linked to T2DM, which are known to frequently coexist with and synergistically increase the CVD risk.

Despite the high prevalence of NAFLD and many epidemiological studies showing correlations between NAFLD and CVD, it is still difficult to unequivocally identify a causal relationship between the two entities [162] and to show that NAFLD is an independent risk factor for CVD [163], given the presence of many comorbidities and confounding factors. In addition, the available studies show great heterogeneity. Also, the genetic variants that predispose to the development of NAFLD have not been linked to the development of atherosclerotic CVD in the absence of general obesity and metabolic syndrome [164].

We are still unclear whether the diagnosis of NAFLD can be used as a tool to improve cardiovascular risk and modify treatment [162]. Lifestyle interventions are recommended by the European clinical guidelines as the best therapeutic option for human NAFLD [9,165]. Moreover, ≥7% weight loss improves steatosis significantly, resulting in lowering of the NAFLD activity score (NAS) [166,167]. On the other hand, only 40% of patients in the above study reached that goal and reduced steatohepatitis, underlining the difficulties in managing NAFLD with lifestyle changes [166]. Nevertheless, reduction of fructose should be recommended for patients with NAFLD along with emerging therapies that can lower the activity of liver enzymes, fibrosis, and inflammation, such as PPARγ inhibitors (pioglitazone), SGLT-2i, and GLP-1 RA, as well as modification in gut microbiota.

## Figures and Tables

**Table 1:** The association between NAFLD and fructose consumption.

Authors (Year)	Study Type	Studies/Participants (N)	Average Duration of Follow-Up	Population	Findings
Zelber-Sagi et al. (2007) [36]	Cross-sectional	1/349	-	Patients with or without NAFLD	The group with diagnosed NAFLD consumed almost twice the amount of SSB. Intake of SSB was significantly associated with an increased risk for NAFLD.
Assy et al. (2008) [37]	Cross-sectional	1/61	36 months	Patients with NAFLD and healthy control group	80% of patients with NAFLD consumed an excessive amount of SSB (more than 50 g/day of added sugar) compared with 20% in healthy controls. SSB consumption was the only independent variable that was able to predict the presence of NAFLD.
Abid et al. (2009) [38]	Prospective	1/90	6 months	Patients with NAFLD and healthy control group	80% of patients with NAFLD had an excessive intake of SSB (>500 cm^3^/day) compared to 17% of healthy controls. Logistic regression analysis showed that SSB consumption is a strong predictor of NAFLD independent of metabolic syndrome and CRP level.
Abdelmalek et al. (2010) [39]	Cross-sectional	1/427	3 months	Patients with NAFLD with none, minimum to moderate, and daily SSB and fruit juices consumption	Increased fructose consumption was associated with hypertriglyceridemia, low HDL-c levels. Daily fructose consumption was associated with lower steatosis grade and higher fibrosis stage. In older adults (age > or = 48 years), daily fructose consumption was associated with increased hepatic inflammation and hepatocyte ballooning.
Maersk et al. (2012) [40]	RCT	1/47	6 months	Overweight patients for 6 months consuming water, milk, diet cola, and regular cola (SSSD)	Milk and diet cola reduced systolic blood pressure by 10–15% compared with regular cola. Daily intake of SSSDs increased accumulation of: liver fat, skeletal muscle fat, visceral fat, blood triglycerides, and total cholesterol, compared with milk, diet cola, and water.
Chiu et al. (2014) [41]	Meta-analysis, systematic review of controlled trials	13/260	More than 7 days	Healthy participants	There was no effect of fructose in isocaloric trials. Increased consumption of fructose in hypercaloric trials increased both IHCL and ALT.
Chung et al. (2014) [42]	Meta-analysis, systematic review of observational and interventional studies	27/1670	6 days to 6 months	Patients with or without NAFLD	Observational studies were rated insufficient because of the high risk of biases and inconsistent study findings. Hypercaloric fructose diet (supplemented by pure fructose) increased liver fat and AST concentrations in healthy men compared with the consumption of a weight-maintenance diet with a low level of evidence. Hypercaloric fructose and glucose diets have similar effects on liver fat and liver enzymes in healthy adults, also with a low level of evidence.
Hochuli et al. (2014) [43]	Randomized crossover	1/34	3 weeks	Healthy young men with medium fructose, high fructose, high sucrose, and high glucose consumption for 3 weeks	Fatty acid synthesis was increased after high fructose consumption and medium fructose consumption compared with high sucrose consumption, high glucose consumption, or baseline. Fasting palmitoylcarnitine was significantly increased after high fructose and high sucrose consumption.
Jin et al. (2014) [44]	RCT	1/24	4 weeks	Overweight patients with average self-reported consumption of at least 3 servings of SSB or fruit juice divided into 2 groups: consuming glucose only beverages and fructose only beverages	There was no significant change in hepatic fat or body weight in the group consuming glucose only or fructose only beverages. In the glucose beverage group, there was significantly improved adipose insulin sensitivity, CRP, and LDL-c oxidation.
Ma et al. (2015) [45]	Cross-sectional	1/2634	3 years	Patients consuming SSB vs. patients who did not consume SSB	Increased incidence of NAFLD was observed in patients with daily consumption of SSB. SSB consumption was positively associated with increased ALT levels.
Schwarz et al. (2015) [29]	Prospective	1/8	9 days	Healthy men on weight-maintaining diets: high in fructose vs. isocaloric diet with complex carbohydrate substituted for fructose	Participants’ weight remained stable. A high fructose diet was associated with higher DNL and higher liver fat in all participants.
Wijarnpreecha et al. (2015) [46]	Meta-analysis, systematic review of cross-sectional and cohort studies	7/4639	6 months to 7 years	Patients consuming a significant amount of either sugar or SSB vs. patients who did not consume SSB	Patients consuming a significant amount of either sugar or SSB have an increased risk of NAFLD.
Chen et al. (2019) [47]	Meta-analysis, systematic review of cross-sectional, case-control and cohort studies	12/35,705	-	Patients consuming low, middle, and high doses of SSB	Consumption of SSB was associated with an increased risk of NAFLD. Consumption of SSB had a dose-dependent effect on the risk of NAFLD.

Abbreviations: ALT—alanine aminotransferase, AST—aspartate aminotransferase, CI—credible interval, CRP—C-reactive protein, DNL—de novo lipogenesis, HDL-c—high-density lipoprotein cholesterol, LDL-c- low-density lipoprotein cholesterol, IHCL—intrahepatocellular lipids, NAFLD—non-alcoholic fatty liver disease, MD—mean difference, OR—odds ratio, RCT—randomized controlled trial, RR—risk ratio, SMD—standardized mean difference, SSB—sugar-sweetened beverages, SSSB—sucrose-sweetened soft drinks.

**Table 2:** Associations between NAFLD and gut microbiota.

Authors (Year)	Study Type	Studies/Participants (N)	Average Duration of Follow-Up	Population	Findings
Raman et al. (2013) [65]	Observational case-control	1/60	-	Obese patients with NAFLD vs. healthy control	In the fecal microbiome of NAFLD patients, there was an over-representation of Lactobacillus species and selected members of phylum Firmicutes and increased fecal ester VOC.
Wang et al. (2016) [61]	Cross-sectional	1/126	-	Non-obese patients with NAFLD vs. healthy control	In non-obese patients with NAFLD, there was lower diversity and a phylum-level change in microbiota compared to healthy control. NAFLD patients had 20% more phylum Bacteroidetes and 24% fewer Firmicutes compared to healthy control.
Shen et al. (2017) [62]	Cross-sectional	1/47	-	Patients with NAFLD vs. healthy control	NAFLD patients had lower gut microbiota diversity than healthy control. In stools of patients with NASH, there were decreased levels of Prevotella, increased levels of Blautia, Lachnospiraceae, Escherichia, Shigella, and Enterobacteriacae compared to patients with NAFLD.
Da Silva et al. (2018) [66]	Cross-sectional	1/67	7 days	Patients with NAFLD, NASH vs. healthy control	In stools of NAFLD patients, Firmicutes, Bacteroidetes were less abundant and Lactobacillaceae more abundant compared to healthy control. NAFLD patients had higher concentrations of fecal propionate and isobutyric acid and serum 2-hydroxybutyrate and L-lactic acid.
Lanthier et al. (2021) [64]	Prospective	1/52	3 months	Obese adults with NAFLD: patients with severe liver steatosis vs. patients with fibrosis	Abundance of fecal Clostridium was significantly decreased with the presence of liver fibrosis and was negatively associated with liver stiffness measurement. Escherichia and Shigella increased fecal abundance was observed in patients with fibrosis compared to patients with severe steatosis without fibrosis.
Li et al. (2021) [63]	Meta-analysis, systematic review	15/1265	-	Adults with NAFLD vs. healthy control group	Stools of patients with NAFLD exhibited an increased abundance of Escherichia, Prevotella, Streptococcus and exhibited a decreased abundance of Coprococcus, Faecalibacterium and Ruminococcus. No significant difference in the abundance of Bacteroides, Bifidobacterium, Blautia, Clostridium, Dorea, Lactobacillus, Parabacteroides, or Roseburia.

Abbreviations: CI—credible interval, NAFLD—non-alcoholic fatty liver disease, NASH—non-alcoholic steatohepatitis, SMD—standardized mean difference, VOC—volatile organic compounds.

**Table 3:** Treatment of NAFLD with microbiome alternation.

Authors (Year)	Study Type	Studies/Participants (N)	Duration of Treatment	Population	Findings
Ma et al. (2013) [68]	Meta-analysis of RCT (probiotic vs. placebo)	4/132	8–24 weeks	Adults with NAFLD	Probiotic therapy was associated with a significant decrease in levels of ALT, AST, total cholesterol, HDL-c, TNF-α, and HOMA-IR.
Eslamparast et al. (2014) [78]	RCT (synbiotic vs. placebo)	1/52	28 weeks	Adults with NAFLD	In patients with NAFLD using synbiotic compared to the placebo group, significantly decreased levels of ALT, AST, GGT, CRP, TNF-α, and fibrosis scores were observed.
Loman et al. (2018) [69]	Meta-analysis of RCT (probiotic or synbiotic or prebiotic vs. placebo)	25/1309	2–28 weeks	Adults with NAFLD	Probiotic/synbiotic/prebiotic therapies were associated with significantly reduced levels of ALT, AST, GGT, total cholesterol, LDL-c, and TAG, but no significant difference in TNF-α and CRP levels.
Bakhshimoghaddam et al. (2018) [77]	RCT (synbiotic vs. control group)	1/102	24 weeks	Adults with NAFLD	Grades of NAFLD determined in ultrasound examination significantly decreased in patients with NAFLD consuming synbiotic, compared to conventional and control groups.
Khan et al. (2019) [70]	Meta-analysis, systematic review of RCT (probiotic or synbiotic vs. placebo)	12/624	8–24 weeks	Adults with NAFLD	Probiotic/synbiotic therapies were associated with a significant reduction in levels of ALT, AST, CRP, and significant improvement in liver fibrosis score.
Sharpton et al. (2019) [71]	Meta-analysis of RCT (probiotic or synbiotic vs. placebo)	21/1252	8–28 weeks	Adults with NAFLD	Probiotic/synbiotic therapies were associated with a significant reduction in levels of ALT and LSM. Usage of probiotics/synbiotics was associated with increased odds of improvement in hepatic steatosis.
Craven et al. (2020) [81]	RCT (allogenic FMT vs. autologous FMT)	1/21	6 months	Adults with NAFLD	There were no significant differences between patients with NAFLD after allogenic FMT and autologous FMT in HOMA-IR or hepatic PDFF. Allogenic FMT in patients with NAFLD with elevated small intestine permeability at baseline caused a significant reduction of small intestine permeability 6 weeks after allogenic FMT.
Pan et al. (2020) [72]	Meta-analysis, systematic review of RCT (probiotic vs. placebo)	19/954		Adults with NAFLD	Probiotic supplementation significantly decreased TNF-α and CRP levels.
Witjes et al. (2020) [82]	RCT (allogenic FMT vs. autologous FMT)	1/21	24 weeks	Adults with NAFLD	Allogenic FMT was associated with modified gut microbiota composition (increased abundance of *ruminococcus*, *eubacterium hallii*, *faecalibacterium*, and *prevotella copri*), decreased levels of GGT, a trend toward improvement in the necro-inflammation score (consisting of both lobular inflammation and hepatocellular ballooning). There was no significant difference in fibrosis score after allogenic FMT.

Abbreviations: ALT—alanine aminotransferase, AST—aspartate aminotransferase, CRP—C-reactive protein, CI—credible interval, GGT—gamma-glutamyl transferase, HDL-c—high-density lipoprotein cholesterol, HOMA-IR—homeostatic model assessment for insulin resistance, LDL-c—low-density lipoprotein cholesterol, LSM—liver stiffness measurement, NAFLD—non-alcoholic fatty liver disease, MD—mean difference, OR—odds ratio, RCT—randomized controlled trial, RR—relative risk, SAT—subcutaneous adipose tissue, SMD—standardized mean difference, T2DM—type 2 diabetes mellitus, TAG—triglyceride, TNF-α—tumor necrosis factor—α, WMD—weighted mean difference.

**Table 4:** NAFLD and T2DM.

Authors (Year)	Study Type	Studies/Participants (N)	Average Duration of Follow-Up	Population	Findings
Lalukka et al. (2016) [85]	Meta-analysis of systematic review, prospective studies	20/122,517	2–20 years	Adults with NAFLD without T2DM	NAFLD predicted the risk of T2DM in all studies. NAFLD predicted the risk of T2DM in all studies with NAFLD diagnosis based on ultrasonography independently of age. NAFLD predicted the risk of T2DM in 12 of 14 studies with NAFLD diagnosis based on liver function tests independently of age or BMI.
Cusi et al. (2017) [93]	Observational	1/204	-	Adults with T1DM and T2DM with or without NAFLD	The prevalence of NAFLD in T1DM patients was low (8.8%) but high in T2DM patients not treated with insulin (75.6%) and treated with insulin (61.7%).
Mantovani et al. (2018) [84]	Meta-analysis of observational studies	19/296,439	at least 5 years	Adults with NAFLD, without T2DM	Patients with NAFLD had a greater risk of T2DM incidence. Patients with advanced NAFLD with fibrosis had an even greater risk of T2DM incidence.
Cho et al. (2019) [94]	Cohort	1/2726	12–135 months	Adults with or without NAFLD or T2DM	Incident and persistent NAFLD increased risk of T2DM development.
Lee et al. (2019) [86]	Cohort	1/6240	4.30 ± 1.91 years	Adults with prediabetes with or without NAFLD from Korea	The prevalence of NAFLD was 45.4%. During follow-up, the incidence of T2DM was 8.1%. Subjects with prediabetes and NAFLD had a higher prevalence of T2DM.
Younossi et al. (2019) [14]	Meta-analysis, systematic review of cross-sectional and longitudinal studies	80/49,419	median 3 years	Adults with T2DM with or without NAFLD and NASH	The global prevalence of NAFLD was 55.5%. The highest prevalence of NAFLD reported in studies from Europe was 68%. The global prevalence of NASH was 37.3%. The prevalence of advanced fibrosis in patients with NAFLD and T2DM was 17%.
Mantovani et al. (2021) [95]	Meta-analysis of prospective studies	33/501,022	at least 1 year	Adults with NAFLD	Patients with NAFLD had a higher risk of incident DM. The risk increased across the severity of NAFLD.

Abbreviations: CI—credible interval, DM—diabetes mellitus, HR—hazard ratio, NAFLD—non-alcoholic fatty liver disease, NASH—non-alcoholic steatohepatitis, RR—risk ratio, T1DM—type 1 diabetes mellitus, T2DM—type 2 diabetes mellitus.

**Table 5:** NAFLD and NASH treatment with antidiabetic drugs.

Authors (Year)	Study Type	Studies/Participants (N)	Duration of Treatment	Population	Findings
Boettcher et al. (2012) [102]	Meta-analysis of RCT (pioglitazone vs. placebo)	4/334	24–96 weeks	T2DM patients with NASH	Pioglitazone treatment was associated with histological improvement of ballooning degeneration, lobular inflammation, and steatosis compared to placebo.
Eguchi et al. (2015) [114]	Prospective (liraglutide vs. lifestyle modification)	1/26	24 weeks	Adults with NASH, BMI ≥ 25 kg/m^2^, with or without T2DM	Liraglutide treatment improved histological features of steatohepatitis and fibrosis in 80% of patients and aminotransferase levels in 78.9% of patients.
Rizvi et al. (2015) [115]	Prospective (liraglutide and metformin therapy vs. metformin therapy)	1/58	8 months	Two groups of T2DM patients with or without NAFLD	Carotid IMT decreased significantly in T2DM patients with NAFLD but not in T2DM patients without NAFLD.
Armstrong et al. (2016) [116]	RCT (liraglutide vs. placebo)	1/52	48 weeks	Adults with NASH, BMI ≥ 25 kg/m^2^, with or without T2DM	Treatment with liraglutide was associated with histological improvement of steatohepatitis.
Armstrong et al. (2016) [117]	RCT (liraglutide vs. placebo)	1/14	12 weeks	Adults with NASH, BMI ≥ 25 kg/m^2^, with or without T2DM	Liraglutide treatment was associated with significant reduction of ALT, increased hepatic insulin sensitivity, suppression of hepatic endogenous glucose production with low-dose insulin, a decrease of hepatic de novo lipogenesis.
Cusi et al. (2016) [103]	RCT (pioglitazone vs. placebo)	1/101	18 months	Patients with prediabetes or T2DM and NASH	Pioglitazone treatment was associated with histological improvement of fibrosis score, reduced hepatic triglyceride content and improved insulin sensitivity of adipose tissue, liver, and muscles.
Feng et al. (2017) [109]	RCT (liraglutide vs. metformin and gliclazide)	1/85	24 weeks	T2DM patients with NAFLD	Liraglutide or metformin monotherapy was associated with greater weight loss, reduction in body fat mass, and improved glucose control compared to gliclazide. Weight loss, fat mass, and waist reduction affected favorably hepatic function IHF decreased significantly after liraglutide.
Seko et al. (2017) [110]	Retrospective study (all patients dulaglutide)	1/15	12 weeks	T2DM patients with biopsy-proven NAFLD	Dulaglutide treatment was associated with significantly decreased BMI, ALT, AST, HbA_1c_ levels.
Cusi et al. (2018) [110]	A post hoc analysis of AWARD program (dulaglutide vs. placebo)	4/1499	6 months	T2DM patients with NAFLD	Dulaglutide treatment was associated with a significant decrease in ALT, AST, and GGT consistent with liver fat reduction.
Kuchay et al. (2018) [108]	RCT (empaglifozin vs. standard treatment)	1/50	20 weeks	T2DM patients with NAFLD	Empagliflozin treatment was associated with significant liver fat reduction and ALT activity improvement compared to the control group.
Shibuya et al. (2018) [105]	RCT (luseogliflozin vs. metformin)	1/32	24 weeks	T2DM patients with NAFLD	Luseogliflozin was associated with significantly greater liver fat reduction than metformin and a significantly greater decrease in VAT and BMI.
Shimizu et al. (2019) [109]	RCT (dapagliflozin vs. control group)	1/57	24 weeks	T2DM patients with NAFLD	Dapagliflozin treatment was associated with a significant decrease in CAP and with a greater significant decrease in ALT and VAT.
Aso et al. (2019) [106]	RCT (dapagliflozin vs. control group)	1/57	24 weeks	T2DM patients with NAFLD	Dapagliflozin was associated with a significant decrease in VAT, SAT, ALT, AST, and GGT.
Yan et al. (2019) [120]	RCT (liraglutide vs. sitagliptin vs. insulin glargine)	1/75	26 weeks	T2DM patients with NAFLD under inadequate glycemic control by metformin	Liraglutide treatment was associated with a significant decrease in MRI-PDFF, VAT, SAT, and body weight. Sitagliptin treatment was associated with a significant decrease in MRI-PDFF, VAT, and body weight.
Hartman et al. (2020) [123]	RCT (tirzepatide vs. dulaglutide vs. placebo)	1/316	26 weeks	T2DM patients with NASH and fibrosis	Tirzepatide treatment was associated with a greater decrease in ALT level than dulaglutide treatment. Adiponectin level increased significantly compared to placebo, but not with dulaglutide therapy.
Kuchay et al. (2020) [121]	RCT (dulaglutide vs. control group)	1/88	24 weeks	T2DM patients with NAFLD	Dulaglutide treatment was associated with a 2.6-fold reduction of LFC and reduction of GGT levels.
Lai et al. (2020) [107]	Prospective, pilot study (empagliflozin vs. placebo)	1/39	24 weeks	T2DM patients with or without NASH	Empagliflozin treatment improved steatosis, ballooning, and fibrosis.
Ghosal et al. (2021) [125]	Meta-analysis, systematic review of RCT (GLP-1 RA vs. placebo)	8/615	12–72 weeks	T2DM patients with NAFLD	GLP-1 RA significantly reduced ALT, AST, GGT levels, LFC, HbA_1c_ levels, and body weight. GLP-1 RA caused significant improvement of NAFLD in biopsy.
Lee et al. (2021) [122]	Meta-analysis, systematic review of RCT (canagliflozin or dapagliflozin vs. placebo)	8/5984	12–18 weeks	T2DM patients with NAFLD	Canagliflozin significantly reduced GGT levels. Dapagliflozin significantly reduced HbA1_c_ levels and HOMA-IR.
Lian et al. (2021) [104]	Meta-analysis of RCT (metformin or liraglutide or pioglitazone vs. placebo)	26/??	12–96 weeks	Patients with NAFLD and with or without T2DM	Pioglitazone had a significant effect on the levels of ALT and AST but was also associated with an increased risk of weight gain and increased BMI. Liraglutide and metformin had significant effects on reducing ALT and AST.
Mantovani et al. (2021) [111]	Meta-analysis of RCT (liraglutide or semaglutide vs. placebo)	22/936	median 26 weeks	Overweight or obese patients with NASH or NAFLD with or without T2DM	Treatment with GLP-1 RA decreased LFC measured by MRI, decreased ALT, GGT, but not AST levels, and greater histological resolution without worsening of liver fibrosis.
Newsome et al. (2021) [112]	RCT (semaglutide vs. placebo)	1/320	72 weeks	Patients with NASH and biopsy confirmed fibrosis	After semaglutide treatment, NASH resolution was achieved in 36–59% of patients with improvement in fibrosis stage in 43% of them.
Ng et al. (2021) [126]	Meta-analysis of RCT (PPARγ agonists or SGLT-2i vs. placebo)	14/??	-	T2DM patients with NAFLD	PPARγ agonists and SGLT-2i significantly reduced steatosis. SGLT-2i resulted in a significantly greater reduction of fibrosis compared to PPARγ.
Song et al. (2021) [13]	Meta-analysis of RCT (liraglutide vs. pioglitazone vs. insulin vs. placebo)	11/535	12–24 weeks	T2DM patients with NAFLD	Liraglutide decreased LFC, BMI, HDL-c, LDL-c, HbA_1c_, TC, and TAG.

Abbreviations: ALT—alanine aminotransferase, AST—aspartate aminotransferase, BMI—body mass index, CAP—controlled attenuation parameter, CI—credible interval, IHF—intrahepatic fat, GLP-1 RA—glucagon-like peptide 1 receptor agonists, GGT—gamma-glutamyl transferase, HbA_1c_—glycated haemoglobin A1c, HDL-c—high density lipoprotein cholesterol, HOMA-IR—homeostatic model assessment for insulin resistance, LDL-c- low density lipoprotein cholesterol, LFC—liver fat content, LSM—liver stiffness measurement, MD—mean difference, mg—milligram, MRI—magnetic resonance imaging, MRI-PDFF—magnetic resonance imaging derived proton density fat fraction, NAFLD—non-alcoholic fatty liver disease, NASH—non-alcoholic steatohepatitis, OR—odds ratio, PPARγ—peroxisome proliferator-activated receptor, RCT—randomized controlled trial, RR—relative risk, SAT—subcutaneous adipose tissue, SGLT-2i—sodium-glucose cotransporter type-2 inhibitors, SMD—standardized mean difference, T2DM—type 2 diabetes mellitus, TAG—triglycerides, TC—total cholesterol, VAT—visceral adipose tissue, WMD—weighted mean difference.

**Table 6:** NAFLD and cardiovascular disease.

Authors (Year)	Study Type	Studies/Participants (N)	Average Duration of Follow-Up	Population	Findings
Targher et al. (2008) [151]	Cross-sectional	1/2103	-	T2DM patients with or without CKD	NAFLD was associated with increased rates of CKD and proliferative/laser-treated retinopathy.
Agarwal et al. (2011) [145]	Retrospective	1/124	-	T2DM adults with or without NAFLD	The prevalence of NAFLD in T2DM patients was 57.2%. In T2DM patients with NAFLD, CAD was more prevalent compared to T2DM patients without NAFLD.
Stepanowa et al. (2012) [139]	Prospective	1/11,613	14 years	Adults with or without NAFLD	NAFLD was associated with a higher risk of CVD. NAFLD was not significantly associated with higher CVD mortality.
Idilman et al. (2014) [146]	Observational	1/273	-	T2DM adults without previous known liver disease	In T2DM patients, NAFLD was associated with significant CAD (≥50 stenosis in CTA).
Kim et al. (2014) [148]	Observational	1/4437	-	T2DM patients with or without NAFLD	The prevalence of NAFLD in T2DM patients was 72.7%. Carotid IMT was significantly higher in T2DM patients with NAFLD and insulin resistance compared to insulin-sensitive T2DM patients without NAFLD and insulin-sensitive T2DM patients with NAFLD.
Li et al. (2014) [152]	Cross-sectional	1/190	-	Adults with diabetes and prediabetes with or without NAFLD	Patients with NAFLD had a higher albumin-to-creatinine ratio. CKD had a higher prevalence in T2DM patients with NAFLD.
Musso et al.(2014) [153]	Meta-analysis of cross-sectional, longitudinal studies	20/29,282	-	T2DM patients with or without NAFLD	NAFLD was associated with an increased risk of prevalent and incident CKD. NASH was associated with a higher prevalence and incidence of CKD than simple steatosis. Advanced fibrosis was associated with a higher prevalence and incidence of CKD than non-advanced fibrosis.
Mellinger et al. (2015) [131]	Prospective cohort	1/3014	3 years	Adults with or without NAFLD	There was no significant association between NAFLD and CVD. NAFLD was associated with CAC and AAC.
Lin et al. (2016) [154]	Cross-sectional	1/5963	-	Adults with NAFLD with or without T2DM	NAFLD was not significantly associated with retinopathy in T2DM patients.
Targher et al. (2016) [159]	Meta-analysis of prospective, retrospective, and observational studies	16/34,043	median period 6.9 years	Adults with or without NAFLD	Patients with NAFLD had a higher risk of MACE than patients without NAFLD.
Unalp-Arida et al. (2016) [140]	Retrospective cohort	1/12,216	6 years	Adults with or without NAFLD	NAFLD was not independently associated with mortality from all causes, including CVD, cancer, or diabetes.
Wu et al. (2016) [138]	Meta-analysis, systematic review of cross-sectional and cohort studies	34/164,494	1.6–26.4 years	Adults with or without NAFLD	NAFLD was associated with increased risk of prevalent and incident CVD, prevalent atherosclerosis, prevalent and incident hypertension, prevalent and incident CAD. NAFLD was not associated with overall and CVD mortality.
Yan et al. (2016) [144]	Observational, retrospective	1/212	-	T2DM patients with or without NAFLD	Patients with NAFLD diagnosed earlier than T2DM had a significantly higher prevalence of CAD and hypertension and lower prevalence of diabetic retinopathy and diabetic peripheral neuropathy compared to T2DM patients with NAFLD diagnosed later than T2DM and T2DM patients without NAFLD. There was no significant difference in the prevalence of diabetic kidney disease.
Zou et al. (2016) [149]	Cross-sectional	1/2646	-	T2DM patients ≥ 40 years old with or without NAFLD	T2DM patients with NAFLD had a significantly higher prevalence of PAD compared with those without NAFLD. The prevalence of NAFLD among T2DM patients was 10.3%. NAFLD was associated with an increased risk of PAD.
Guo et al. (2017) [147]	Cross-sectional	1/8571	-	T2DM patients with or without NAFLD	The prevalence of carotid and lower limb plaque, as well as carotid and lower limb stenosis, was significantly higher in T2DM patients with NAFLD than in T2DM patients without NAFLD. There was no significant difference between T2DM patients with or without NAFLD in carotid IMT.
Yoshitaka et al. (2017) [136]	Cohort	1/1674	6 years	Overweight and non-overweight patients with or without NAFLD	NAFLD was associated with a higher risk of CVD incidents in non-overweight patients with NAFLD.
Kapuria et al. (2018) [129]	Meta-analysis, systematic review of cross-sectional and cohort studies	12/42,410	-	Adults with or without NAFLD	NAFLD was associated with a higher CAC score compared to adults without NAFLD.
Zhou et al. (2018) [130]	Meta-analysis, systematic review of cross-sectional, case-control, and cohort studies	26/83,395	-	Adults with or without NAFLD	NAFLD was associated with a higher risk of increased carotid IMT, arterial stiffness, coronary artery calcification, and endothelial disfunction.
Zhou et al. (2018) [143]	Meta-analysis of cross-sectional, cohort studies	11/8346	At least 5 years	T1DM and T2DM adults with or without NAFLD	T2DM patients with diagnosed NAFLD had a 2 times higher risk for CVD compared with patients without NAFLD.
Afarideh et al. (2019) [150]	Case-control	1/935	-	T2DM patients with at least one microvascular complication vs. T2DM patients control group	Diabetic retinopathy and DKD were inversely associated with the presence of NAFLD. The subgroup of NAFLD with elevated liver enzymes had lower odds of having diabetic peripheral neuropathy.
Alexander et al. (2019) [141]	Cohort	1/120,795	mean 2.1–5.5 years	Adults with NAFLD	After adjustment for established cardiovascular risk factors, NAFLD was not associated with AMI or stroke risk.
Lee et al. (2020) [160]	Cohort	1/1120	6–8 years	T2DM patients with or without NAFLD	NAFLD was significantly associated with atherosclerosis progression.
Mann et al. (2020) [137]	Retrospective cohort	1/26,539	14 years after discharge	Patients with or without NAFLD	Patients with NAFLD without cirrhosis and NAFLD with cirrhosis had higher mortality compared to controls.
Shao et al. (2020) [135]	Cross-sectional	1/543	-	Obese patients with NAFLD vs. non-obese patients with NAFLD	Predictive factors of subclinical atherosclerosis in all patients with NAFLD were age increased per 10 years and liver stiffness. LFC was an additional predictor in obese patients with NAFLD.
Greco et al. (2021) [161]	Meta-analysis, systematic review of cross-sectional studies	13/9614	-	T1DM and T2DM patients with or without NAFLD	Diabetic peripheral neuropathy prevalence was significantly higher in T2DM patients with NAFLD compared to T2DM patients without NAFLD, but not in T1DM patients with NAFLD.
Meyersohn et al. (2021) [133]	Cohort	1/3756	25 months	Symptomatic patients without previous diagnosed CAD	NAFLD was associated with MACE independently of other cardiovascular risk factors or extent of CAD.
Lee et al. (2021) [142]	Cohort	1/8,962,813	median 10.1 years	Adults with or without NAFLD	NAFLD and MAFLD were associated with significantly higher risk for CVD events.

Abbreviations: AAC—abdominal artery calcium, AMI—acute myocardial infarction, AOR—adjusted odds ratio, CAC—coronary artery calcium, CAD—coronary artery disease, CI—credible interval, CKD—chronic kidney disease, CTA—computed tomography angiography, CVD—cardiovascular disease, HR—hazard ratio, DKD—diabetic kidney disease, IMT—intima-media thickness, LFC—liver fat content, MACE—major adverse cardiovascular events, MAFLD—metabolic associated fatty liver disease, NAFLD—non-alcoholic fatty liver disease, OR—odds ratio, PAD—peripheral artery disease, T1DM—type 1 diabetes mellitus, T2DM—type 2 diabetes mellitus.

## Data Availability

No new data were created or analyzed in this study. Data sharing is not applicable to this article.
